# Practice change intervention to improve antenatal care addressing alcohol consumption during pregnancy: a randomised stepped-wedge controlled trial

**DOI:** 10.1186/s12884-022-04646-7

**Published:** 2022-04-21

**Authors:** Emma Doherty, Melanie Kingsland, Elizabeth J. Elliott, Belinda Tully, Luke Wolfenden, Adrian Dunlop, Ian Symonds, John Attia, Sarah Ward, Mandy Hunter, Carol Azzopardi, Chris Rissel, Karen Gillham, Tracey W. Tsang, Penny Reeves, John Wiggers

**Affiliations:** 1 Population Health, Hunter New England Local Health District, Wallsend, New South Wales 2287 Australia; 2 School of Medicine and Public Health, College of Health, Medicine and Wellbeing, The University of Newcastle, Callaghan, New South Wales 2308 Australia; 3 Hunter Medical Research Institute, New Lambton Heights, New South Wales 2305 Australia; 4 Faculty of Medicine and Health and Discipline of Child and Adolescent Health, The University of Sydney, Sydney, New South Wales 2006 Australia; 5 Sydney Children’s Hospital Network, Kids’ Research Institute, Westmead, New South Wales 2145 Australia; 6 Drug and Alcohol Clinical Services, Hunter New England Local Health District, Newcastle, New South Wales 2302 Australia; 7 Adelaide Medical School, The University of Adelaide, Adelaide, South Australia 5005 Australia; 8 Foundation for Alcohol Research and Education, Deakin, Australian Capital Territory 2600 Australia; 9 Maternity and Gynaecology John Hunter Hospital, New Lambton Heights, New South Wales 2305 Australia; 10 College of Medicine and Public Health, Flinders University, Casuarina, Northern Territory 0909 Australia

**Keywords:** Antenatal care, Alcohol, Pregnancy, Clinical practice change, Implementation, Stepped-wedge trial, Outcomes

## Abstract

**Background:**

Clinical guideline recommendations for addressing alcohol consumption during pregnancy are sub-optimally implemented and limited evidence exists to inform practice improvements. The aim of this study was to estimate the effectiveness of a practice change intervention in improving the provision of antenatal care addressing alcohol consumption during pregnancy in public maternity services.

**Methods:**

A randomised stepped-wedge controlled trial was undertaken with all public maternity services in three sectors (one urban, two regional/rural) of a single local health district in New South Wales, Australia. All antenatal care providers were subject to a seven-month multi-strategy intervention to support the introduction of a recommended model of care. For 35 months (July 2017 – May 2020) outcome data were collected from randomly selected women post an initial, 27–28 weeks and 35–36 weeks gestation antenatal visit. Logistic regression models assessed intervention effectiveness.

**Results:**

Five thousand six hundred ninety-four interviews/online questionnaires were completed by pregnant women. The intervention was effective in increasing women’s reported receipt of: assessment of alcohol consumption (OR: 2.63; 95% CI: 2.26–3.05; *p* < 0.001), advice not to consume alcohol during pregnancy and of potential risks (OR: 2.07; 95% CI: 1.78–2.41; *p* < 0.001), complete care relevant to alcohol risk level (advice and referral) (OR: 2.10; 95% CI: 1.80–2.44; *p* < 0.001) and all guideline elements relevant to alcohol risk level (assessment, advice and referral) (OR: 2.32; 95% CI: 1.94–2.76; *p* < 0.001). Greater intervention effects were found at the 27–28 and 35–36 weeks gestation visits compared with the initial antenatal visit. No differences by sector were found. Almost all women (98.8%) reported that the model of care was acceptable.

**Conclusions:**

The practice change intervention improved the provision of antenatal care addressing alcohol consumption during pregnancy in public maternity services. Future research could explore the characteristics of pregnant women and maternity services associated with intervention effectiveness as well as the sustainment of care practices over time to inform the need for, and development of, further tailored practice change support.

**Trial registration:**

Australian and New Zealand Clinical Trials Registry (Registration number: ACTRN12617000882325; Registration date: 16/06/2017) https://www.anzctr.org.au/Trial/Registration/TrialReview.aspx?id=372985&isReview=true

## Background

Alcohol consumption during pregnancy is associated with adverse outcomes for the exposed child, including birth defects, developmental delays and Fetal Alcohol Spectrum Disorder [1–3]. It also contributes to pregnancy complications and poor obstetric outcomes, such as impaired placental blood flow, intrauterine growth restriction and stillbirth [4–6]. As there is no determined threshold for the safe consumption of alcohol during pregnancy, many countries have produced guidelines that recommend pregnant women do not consume alcohol [7]. Despite this, the global prevalence of alcohol consumption at any time during pregnancy has been estimated at 9.8% with notably higher rates of consumption reported in Ireland (60.4%), Denmark (45.8%), United Kingdom (41.3%) and Australia (35.6%) [8].

Systematic reviews support the effectiveness of psychosocial and brief interventions, including those delivered by health professionals, in increasing abstinence and reducing levels of alcohol consumption during pregnancy [9–11]. Consistent with such evidence, international [12] and Australian national [13] clinical guidelines recommend at the initial antenatal care visit and in subsequent visits throughout pregnancy all women receive: i) assessment of alcohol consumption using a validated tool; ii) brief advice that it is safest not to consume alcohol during pregnancy and an explanation of the potential risks associated with consumption; and iii) referral to specialist services for further support if required.

Despite the existence of such guidelines, provision of the recommended care elements is highly variable in public maternity services [14–18]. Whilst the majority of women report being asked about their alcohol consumption at some point during their pregnancy (51–97%) [14–17], less than half report being: assessed using a validated tool (42%) [18]; advised about alcohol consumption (11–35%) [15, 17]; and referred to further support if required (10–50%) [14, 17]. Further, just over a quarter (28%) of pregnant women report receiving all guideline care elements (assessment, advice and referral) relevant to their alcohol risk level at the initial antenatal visit and 4% at subsequent antenatal visits [17]. The provision of such care has been reported to vary across maternity services, with larger and urban based services associated with lower levels of care provision [17, 19].

A variety of barriers may impede maternity services from implementing these guideline recommendations for addressing alcohol consumption during pregnancy as part of routine antenatal care. Such barriers have been reported at the individual level for both the health professionals delivering care (e.g. lack of knowledge and a perception that women may not find care acceptable) [18, 20, 21] and the managers responsible for the implementation of the clinical guideline in their antenatal service (e.g. stress) [20] as well as more broadly at the organisational level (e.g. lack of environmental systems and resources to prompt care) [20, 22]. Implementation strategies that have demonstrated effectiveness in increasing evidence-based practice in healthcare generally, and maternity services specifically, such as educational meetings [23, 24], local opinion leaders [25–27], audit and feedback [28–30] and electronic prompts [31], may overcome such barriers to care provision. However, given the variable results reported in systematic reviews on the effectiveness of such strategies for a variety of care practices (absolute improvement range: 0–20%) [23–32], it is recommended that strategy development be guided by an implementation framework and tailored to local context and barriers in order to maximise intervention effectiveness [33, 34]. Interventions that have been developed in this way have been shown to yield improvements in care provision in the range of 9 to 47% [35–37].

Only one controlled trial to date has assessed the effectiveness of implementation strategies in improving antenatal care addressing alcohol consumption. The 2013 trial conducted with Obstetrics and Gynaecology Units in four Italian public hospitals found that a significantly greater proportion of women who attended a hospital that was provided with training and action research support, received ‘correct’ advice from a midwife (53%), compared with women who received advice from a midwife at a control hospital (20%; RR: 2.66, 95% CI: 1.27–5.56) [38]. The trial, however, was non-randomised, did not report or adjust for baseline rates of care delivery and had a small sample size for the advice outcome (*N* = 67). To address this evidence gap, we conducted a study to examine the effectiveness of a multi-strategy practice change intervention in improving antenatal care addressing alcohol consumption during pregnancy.

## Methods

### Aim

The aim of this study was to estimate the effectiveness of a practice change intervention in increasing the provision of guideline recommended antenatal care (assessment, advice and referral) addressing alcohol consumption during pregnancy by public maternity services. The differential effect of the intervention on care provision by type of antenatal visit and sector, and pregnant women’s acceptability of the model of care implemented were also examined.

### Study design and setting

**Fig. 1:**
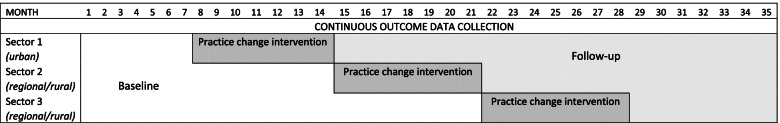
Data collection and intervention timeline for the randomised stepped-wedge controlled trial

A randomised stepped-wedge controlled trial was conducted in all public maternity services in three geographically and administratively defined sectors (clusters) of the Hunter New England Local Health District (HNELHD) in New South Wales, Australia. The three sectors were selected because they represented a mixture of areas and were of sufficient size. A seven-month practice change intervention was delivered sequentially in each of the sectors. Data were collected continuously across all sectors for 35 months (July 2017 to May 2020) with the primary outcomes determined by comparing practice change between baseline and follow-up periods for the three sectors combined (see Fig. 1). The maternity services provide antenatal care to 6100 women annually (70% of births in the district) in one major city (Sector One: 4300 births per annum) and two regional/rural areas (Sectors Two and Three: 1200 and 600 births respectively) [39].

The study was registered with the Australian and New Zealand Clinical Trials Registry (registration number: ACTRN12617000882325; registration date: 16/06/2017). Reporting of this study is in accordance with the Consolidated Standards of Reporting Trials (CONSORT) statement for stepped-wedge cluster randomised trials. We obtained ethics approval before we began the study (HNELHD: 16/11/16/4.07, 16/10/19/5.15; The University of Newcastle: H-2017-0032, H-2016-0422; and Aboriginal Health and Medical Research Council: 1236/16). Study methods are further outlined in the published protocol [40].

### Random allocation and blinding

An independent statistician randomly allocated the order of intervention delivery to the three participating sectors. Study personnel randomly selected women to participate in data collection and those involved in collecting outcome data were blind to intervention order. All randomisations were non-stratified and conducted using a computerised random-number generator. As the intervention changes practice, we could not blind antenatal providers to the intervention.

### Participant eligibility and recruitment

#### Maternity services and providers

All maternity services within the three sectors received the practice change intervention. The types of services included: hospital and community-based midwifery clinics; hospital medical clinics; midwifery continuity of care group practices; Aboriginal Maternal and Infant Health Services (AMIHS); and specialist services caring for women with complex pregnancies or social vulnerabilities. All antenatal care providers in these services were eligible to receive the implementation strategies, including midwifery and medical staff and Aboriginal Health Workers (AHWs). Clinicians who were not the primary providers of antenatal care (e.g. social workers) were not targeted for the intervention.

#### Pregnant women

All women who attended a participating maternity service had the potential to receive the recommended model of care. During the 35-month study period, women were eligible to participate in study interviews/online questionnaires if they: attended an initial antenatal visit or 27–28 weeks gestation visit or 35–36 weeks gestation visit with a participating public maternity service in the preceding week; were 18 years or older; were 12 to 37 weeks gestation; had a sufficient level of English; and were mentally and physically capable of completing the interview/online questionnaire. Women were ineligible for data collection if: receiving majority of antenatal care through a private provider; had already given birth; had a negative pregnancy outcome; were already selected to participate in the study in the past four weeks; or previously declined participation.

#### Procedure for recruiting women for interviews/online questionnaires

Extracts from the maternity service’s medical record and appointment systems were used to randomly generate a weekly sample of 105 eligible women across the three sectors (initial visit: 30 women; 27–28 weeks gestation visit: 30 women; 35–36 weeks gestation visit: 45 women). Sampled women were first mailed an information statement outlining the purpose of the study. One week later, non-Aboriginal women were called to invite participation in a telephone interview with online mode offered if the telephone interview was declined. Based on advice received regarding a culturally appropriate survey approach for Australia’s First Nations peoples, women identifying as Aboriginal and/or Torres Strait Islander (the term Aboriginal will be used from this point when referring to Aboriginal and/or Torres Strait Islander peoples or organisations) and/or women attending AMIHS were sent a text message offering either telephone interview or online modes. Women received up to 10 telephone contact attempts within a two-week period with the same time limit applied for completion of the online questionnaire.

### Intervention

#### Model of care addressing alcohol consumption during pregnancy

**Fig. 2:**
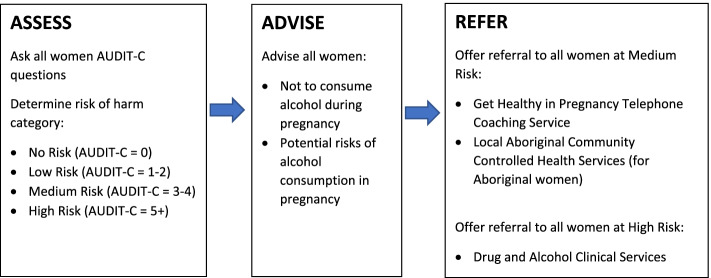
Recommended model of care for addressing alcohol consumption at the initial antenatal visit, 27–29 weeks gestation visit and 35–37 weeks gestation visit

A model of care consistent with systematic review evidence of effective interventions in reducing alcohol consumption during pregnancy [9–11] and international [12] and Australian national [13] clinical guideline recommendations was implemented. The model of care was delivered to women who attended an initial antenatal visit, a 27–29 weeks gestation visit and 35–37 weeks gestation visit. Women attend their initial antenatal visit with the public maternity service at a mean gestation of 19 weeks. The 27–29 and 35–37 weeks gestation visits were selected by maternity services as they are the only two subsequent visits that all women are scheduled to attend. The recommended model of care consisted of three key elements (see Fig. 2):

1. Assess: Assessment of all women’s alcohol consumption using the three item Alcohol Use Disorders Identification Test - Consumption (AUDIT-C) tool [41]. The total score was used to assign an alcohol risk of harm category: No Risk (score = 0); Low Risk (score: 1–2); Medium Risk (score: 3–4); and High Risk (score: 5+) [42].
2. Advise: Provision of two components of advice to all women: i) that it is safest not to consume alcohol during pregnancy; and, ii) explanation of the potential risks associated with alcohol consumption during pregnancy.
3. Refer: Offer of referral to the free government Get Healthy in Pregnancy telephone coaching service [43] to all women assessed as being at Medium Risk, with Aboriginal women also offered referral to counselling at Aboriginal Community Controlled Health Services. Offer of referral to all women at High Risk to the Drug and Alcohol service provided by the health district. Follow-up of women who had previously accepted a referral to an abovementioned service at the 27–29 and 35–37 weeks gestation visits.

#### Implementation strategies

The implementation strategies were developed through a staged process. First, antenatal provider and manager barriers to the implementation of the recommended model of care were explored using a quantitative online questionnaire based on the Theoretical Domains Framework (TDF) [44, 45]. The TDF consolidates constructs from 33 behaviour change theories and is one of the most commonly applied frameworks in implementation science as it incorporates constructs at both the individual (e.g. knowledge) and broader environmental context (e.g. resources) levels. It is used as a planning tool in intervention development to identify factors (i.e. barriers and enablers) influencing behaviour and subsequently guide selection of the most appropriate behaviour change techniques [44, 45]. Next, implementation strategies that incorporated TDF behaviour change techniques for the identified barriers were chosen based on a review of the literature and in consultation with experts in implementation science, clinical practice change, health service research and treatment of alcohol harms. The application of the selected implementation strategies in maternity services were then developed through consultations with key antenatal providers and managers in each sector. The content and delivery of strategies to the local context was also tailored to each sector’s usual processes. Lastly, cultural appropriateness was embedded into the implementation strategies through consultations with Aboriginal health staff, local community members and organisations, as well as focus groups with Aboriginal women who had recently attended a participating maternity service. Further detail on the development of the implementation strategies, including the findings of the antenatal provider and manager questionnaires, have been published elsewhere [20, 40]. All strategies other than the dedicated Clinical Midwife Educator (CME) as the local opinion leader and academic detailing were implemented with the potential and intention that they continue to be implemented post the seven-month intervention period due to their organisational and systems focus (see Table 1 for a description of the implementation strategies).

**Table 1:** Implementation strategies

Implementation strategy	Description
Leadership/ managerial supervision [25]	Meetings were held every 2 months with maternity service management to elicit operational support for the practice change. Management demonstrated leadership by distributing key documentation and communications to staff, being present at training sessions, and by monitoring performance measures relating to the practice change.
Local clinical practice guidelines [32]	A service level guideline and procedure document that outlined the model of care was uploaded onto the health service’s policy and guidelines directory and disseminated by managers to all staff via email and hard copies were placed in staff common areas.
Electronic prompt and reminders [31]	Modifications were made to the existing point-of-care electronic medical record system used by maternity services. Changes to the system included: an electronic prompt for care at the three antenatal visits; standardised assessment of alcohol consumption using AUDIT-C, auto-calculation of AUDIT-C risk; brief advice scripts based on risk of harm category; and prompts for referral services. Antenatal providers were also provided with written point of care prompts, including stickers in hard-copy medical charts, and assessment prompts printed on a handheld ‘pregnancy wheel’ used by antenatal providers to determine gestation.
Local opinion leaders/ champions [25–27]	A dedicated CME was appointed in each sector to provide individual, team and service level support in the uptake of the recommended model of care. The CME was responsible for delivering and monitoring the implementation strategies and was appointed based on their ability to engage staff and model the required behaviours. Additional local antenatal clinical leaders were engaged to provide encouragement and demonstration of required behaviours in each maternity service as required.
Educational meetings and educational materials [23, 24]	A 30-min online training module and a series of face-to-face sessions (including a mix of didactic, interactive, case-study, group and one-on-one sessions) were facilitated by the CME and a content expert. Antenatal providers were also given written educational resources to support the model of care, including standard drinks charts and fact sheets on the harm of alcohol consumption during pregnancy.
Academic detailing, including audit and feedback [28–30]	Data that were collected from medical records and interviews/online questionnaires with pregnant women who recently attended a service were fed back to antenatal providers by the CME. The CME supported providers to develop action plans in response to the data for each of the guideline elements (assessment, advice and referral). Data on women’s reported acceptability of the model of care was also fed back to services.
Monitoring and accountability for performance [29]	Performance measures for the model of care for addressing alcohol consumption during pregnancy were included in managers’ existing monitoring and accountability frameworks, including measures in service-level operational plans and on the health district’s performance platform. Managers were supported in interpreting and disseminating the data to their staff through usual communication mechanisms, such as team meetings and email.

### Control group

Before the intervention, each of the three sectors provided antenatal care addressing alcohol consumption during pregnancy as usual. Such care varied between maternity services as no local procedures were in place. The only guidance to provide care for alcohol consumption in antenatal visits prior to the intervention was a single non-validated question in the medical record at the initial antenatal visit.

### Primary and secondary outcomes

The primary outcomes of the study are the proportion of pregnant women at the initial, 27–28 weeks gestation and 35–36 weeks gestation antenatal visits who report receipt of: i) assessment for alcohol consumption using the AUDIT-C; ii) brief advice related to alcohol consumption during pregnancy; iii) care relative to alcohol risk level (advice and referral); and iv) assessment for alcohol consumption using the AUDIT-C and care relative to alcohol risk level (advice and referral). Secondary outcomes reported in this paper are the effects of the intervention by antenatal visit and sector and pregnant women’s acceptability of the model of care.

### Data collection procedures

Data were collected through women’s self-report interviews/online questionnaires as it is subject to less response bias than health-professional self-report of clinical adherence and can provide complete outcome data unlike medical records [46]. Questions used in the interviews/online questionnaires were developed based on previous studies conducted with pregnant women about their consumption of alcohol [47] and self-report of receipt of healthcare [16, 48]. Data regarding receipt of antenatal care addressing alcohol consumption and the demographics of women were collected through the interviews/online questionnaires. The telephone interviews were conducted by trained female interviewers who were independent from the maternity services and project team. The interview and questionnaire were reviewed for cultural appropriateness by Aboriginal women and pilot tested prior to the study commencing. Additional data regarding women’s demographics and service characteristics were obtained from the district’s medical record and appointment systems and project logs.

### Measures

#### Receipt of antenatal care addressing alcohol consumption during pregnancy

Women were asked whether their antenatal care providers assessed their alcohol consumption during the antenatal visit and, if so, whether this was through questions consistent with the AUDIT-C tool (were you asked: how often you currently consume alcohol; number of standard drinks on a typical drinking day; and occasions of consuming 5 or more standard drinks?) (yes, no, don’t know). All women were asked whether they were: advised that it is safest not to consume alcohol during pregnancy; advised of the potential risks of consuming alcohol during pregnancy; and offered a referral for further support. Women who were completing an interview/online questionnaire for a 27–28 or 35–36 weeks gestation visit were also asked if they had accepted a referral for alcohol consumption in a previous antenatal visit and, if so, whether progress of the referral was followed-up.

#### Acceptability of the model of care addressing alcohol consumption during pregnancy

During the intervention follow-up period, women’s acceptability of alcohol consumption being addressed as part of routine antenatal care was assessed using a 5-point Likert scale (possible responses: strongly agree, agree, unsure, disagree, strongly disagree). Women reported their acceptability overall and for each care element received in the antenatal visit, including: being asked about alcohol consumption, being advised that it is safest not to consume alcohol during pregnancy and being advised about the potential risks of alcohol consumption.

#### Demographics of pregnant women

Women reported in the interview/online questionnaire their: age, Aboriginal origin, education, employment, marital status and gravidity. Information on woman’s postcode and allocated model of antenatal care were collected from the medical record and appointment systems. All women were asked to report their alcohol consumption using the AUDIT-C tool [41].

### Sample size and power calculations

Data for estimating the intra-class correlation co-efficient (ICC) could not be derived from previous cluster randomised trials. Given that the outcomes within clusters were not expected to be highly correlated and the magnitude of outcomes between clusters different, an ICC of 0.01 was selected. Based on this, it was predicted that 200 completed interviews/online questionnaires per month would provide 80% power to detect an absolute increase in care provision of 15% (based on a conservative estimate of 50% care provision at baseline) in at least one of the four primary outcomes at a 1.25% significance threshold (Bonferroni adjusted for the four primary outcomes). Eighty percent power was chosen as there were only three sectors (clusters) that were assessed as suitable for the trial.

### Statistical analysis

Statistical analyses were undertaken using SAS version 9.3 [49]. Condensed response categories were created for pregnant women’s demographics. We grouped total AUDIT-C scores according to national guidelines [42]. Women’s reported acceptability of each of the care elements was dichotomised into ‘acceptable’ (strongly agree and agree) and ‘not acceptable’ (strongly disagree, disagree and unsure). Aboriginal women’s acceptability of the model of care was also examined separately given the embedding of cultural inclusion into the practice change intervention.

Response options to the receipt of care questions were dichotomised (yes/no) with responses of ‘don’t know’ coded as ‘no’. The following primary outcome variables were created:

- Assessment of alcohol consumption: reported receipt of a question consistent with the first AUDIT-C question (for women who reported in the interview/online questionnaire an AUDIT-C score of 0) and reported receipt of all three questions consistent with the AUDIT-C (for women with AUDIT-C ≥ 1).
- Brief advice related to alcohol consumption during pregnancy: reported receipt of advice that it is safest not to consume alcohol during pregnancy and of the potential risks associated with alcohol consumption during pregnancy (all women).
- Complete care (brief advice and referral) relative to level of alcohol risk: reported receipt of complete advice (all women) and referral offered or followed-up (for AUDIT-C ≥ 3).
- Assessment of alcohol consumption using the AUDIT-C and complete care (brief advice and referral) relative to level of alcohol risk: reported assessment via AUDIT-C (all women) and complete advice (all women) and referral offered or followed-up (for AUDIT-C ≥ 3).

Descriptive statistics were used to describe women’s demographics and reported receipt and acceptability of the model of care. To assess the change in receipt of care from baseline to follow-up, logistic regression models were used. For each outcome, the model included a period term (fixed effect; baseline - follow-up difference) and was adjusted for sector (fixed effect; clusters one, two and three), antenatal visit (fixed effect; initial visit, 27–28 weeks gestation, 35–36 weeks gestation) and time (fixed effect; month of antenatal visit). To explore the intervention effect over time within and between antenatal visit types an interaction term (period term x antenatal visit) was included in the above models, with the between group analysis combining subsequent antenatal visits (27–28 weeks and 35–36 weeks gestation visits) for comparison with the initial antenatal visit. We also explored the intervention effect over time within and between sectors by including an interaction term (period term x sector) into the above models. We summarise the effects of the intervention by Odds Ratios (ORs) with their 95% Confidence Intervals (CIs) and significance levels.

### Deviation from protocol [40]

The practice change intervention was delivered at seven monthly intervals instead of the planned six months resulting in data being collected for 35 months instead of 34 months. The number of women sampled per week was increased from 72 to 105 in order to meet the required number of interviews/online questionnaires to power the study. More women were sampled at 35–36 weeks gestation (45 per week, compared with 30 per week for other visits) to account for the larger number of women at this time point who became ineligible between sampling and data collection as they had given birth. Formal meetings with management were held bi-monthly instead of monthly with informal communication occurring between meetings to enable quicker feedback on the implementation of the intervention. Ninety-eight percent of antenatal providers received training during the intervention period instead of the planned 100%.

## Results

### Maternity services and providers

All 28 antenatal care teams in the three sectors participated in the study: 13 hospital and community-based midwifery clinics; five hospital medical clinics; five AMIHS; three midwifery continuity of care group practices; one specialist service caring for women with complex pregnancies; and one specialist service caring for women with social vulnerabilities. Three hundred and twenty-nine antenatal care providers (233 midwifery; 82 medical; and 14 AHWs) delivered antenatal care during the intervention period in the three sectors.

### Pregnant women

**Fig. 3:**
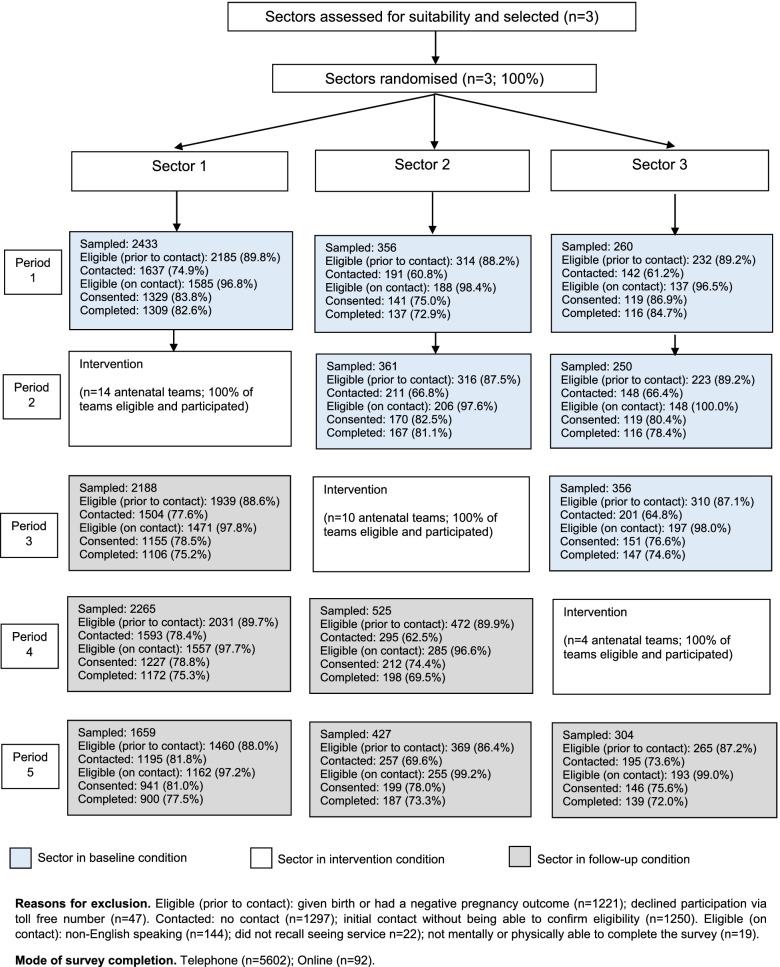
CONSORT Flowchart

Of 11,384 women who were selected to participate in data collection, 10,116 (88.9%) were deemed eligible and of these, 7571 (74.8%) were contacted within the two-week contact period. Of the 7386 women who were eligible on contact, 5909 (80.0%) consented to participate and 5694 (77.1%) completed an interview/online questionnaire (see Fig. 3). Most participants were not Aboriginal (94.7%), had completed at least a technical certificate or diploma (72.6%) and were employed (70.9%) (see Table 2).

**Table 2:** Pregnant women’s demographics

	Baseline (***N*** = 1992)	Follow-up (***N*** = 3702)	Total (***N*** = 5694)
	n (%)	n (%)	n (%)
Age			
Mean (SD)	29.3 (5.3)	30.2 (5.2)	29.9 (5.2)
Aboriginal, or Torres Strait Islander, or both	122 (6.1%)	182 (4.9%)	304 (5.3%)
Highest education level completed			
Completed high school or less	590 (29.6%)	960 (25.9%)	1550 (27.2%)
Completed technical certificate or diploma	740 (37.1%)	1299 (35.1%)	2039 (35.8%)
Completed university or college degree or higher	660 (33.1%)	1438 (38.8%)	2098 (36.8%)
Employment status			
Employed full time	647 (32.5%)	1417 (38.3%)	2064 (36.2%)
Employed part time or casual	685 (34.4%)	1293 (34.9%)	1978 (34.7%)
Home duties	348 (17.5%)	506 (13.7%)	854 (15.0%)
Student	60 (3.0%)	77 (2.1%)	137 (2.4%)
Not employed	251 (12.6%)	407 (11.0%)	658 (11.6%)
Marital status			
Married or defacto relationship	1711 (85.9%)	3289 (88.8%)	5000 (87.8%)
Geographic remoteness			
Major city	1149 (57.7%)	2826 (76.3%)	3975 (69.8%)
Regional and rural	843 (42.3%)	875 (23.6%)	1718 (30.2%)
Area index of disadvantage			
Most disadvantaged	1253 (62.9%)	1913 (51.7%)	3166 (55.6%)
Least disadvantaged	739 (37.1%)	1788 (48.3%)	2527 (44.4%)
First Pregnancy	818 (41.1%)	1476 (39.9%)	2294 (40.3%)
Allocated model of antenatal care			
Low risk	1233 (61.9%)	2273 (61.4%)	3506 (61.6%)
High risk	759 (38.1%)	1420 (38.4%)	2179 (38.3%)

Access/Remoteness Index of Australia [50] was used for categorising Geographic remoteness and Index of Relative Socio-Economic Disadvantage (IRSD) [51] for Area index of disadvantage

Demographic variables are missing data from between 1 and 9 participants

### Receipt of antenatal care addressing alcohol consumption during pregnancy

As shown in Table 3, the odds of women reporting receipt of an assessment consistent with the AUDIT-C (baseline: 28.4% vs follow-up: 40.6%; OR: 2.63; 95% CI: 2.26–3.05) and receipt of complete brief advice (baseline: 18.7% vs follow-up: 26.7%; OR: 2.07; 95% CI: 1.78–2.41) was significantly greater at follow-up for the three sectors combined. Significant intervention effects were also found for receipt of complete care (advice and referral) (baseline: 18.5% vs follow-up: 26.6%; OR: 2.10; 95% CI: 1.80–2.44) and receipt of all guideline care elements (assessment and complete care) (baseline: 12.6% vs follow-up: 19.4%; OR: 2.32; 95% CI: 1.94–2.76).

**Table 3:** Receipt of antenatal care addressing alcohol consumption during pregnancy overall and by type of antenatal visit

	**Initial antenatal visit**						**27–28 weeks gestation visit**							
**Model of care element**	**Baseline** ***N*** **= 682**		**Follow-up** ***N*** **= 1161**		**OR (95% CI)**	***p*-value**	**Baseline** ***N*** **= 670**		**Follow-up** ***N*** **= 1139**		**OR (95% CI)**	***p*-value**		
	**n**	**%**	**n**	**%**			**n**	**%**	**n**	**%**				
Assessment of alcohol consumption (via AUDIT-C)	451	66.1%	821	70.7%	1.45 (1.17; 1.79)	< 0.001	67	10.0%	318	27.9%	4.17 (3.11; 5.59)	< 0.001		
Complete brief advice (safest not to consume and potential risks)	245	35.9%	478	41.2%	1.50 (1.22; 1.84)	< 0.001	81	12.1%	245	21.5%	2.41 (1.82; 3.19)	< 0.001		
Advice safest not to consume	447	65.5%	853	73.4%	1.77 (1.43; 2.19)	< 0.001	132	19.7%	424	37.2%	2.99 (2.36; 3.78)	< 0.001		
Advice on potential risks	268	39.3%	508	43.7%	1.43 (1.17; 1.75)	< 0.001	150	22.4%	348	30.5%	1.83 (1.45; 2.30)	< 0.001		
Complete care relative to level of alcohol risk (complete brief advice and referral)	243	35.6%	477	41.1%	1.51 (1.23; 1.86)	< 0.001	81	12.1%	244	21.4%	2.40 (1.81; 3.18)	< 0.001		
Assessment of alcohol consumption (via AUDIT-C) and complete care relative to level of alcohol risk	192	28.2%	392	33.8%	1.64 (1.32; 2.04)	< 0.001	36	5.4%	151	13.3%	3.43 (2.33; 5.05)	< 0.001		
	**35–36 weeks gestation visit**						**Total**						**Between group differences**	
**Model of care element**	**Baseline** ***N*** **= 637**		**Follow-up** ***N*** **= 1398**		**OR (95% CI)**	***p*-value**	**Baseline** ***N*** **= 1989**		**Follow-up** ***N*** **= 3698**		**OR (95% CI)**	***p*-value**	**Initial vs subsequent antenatal visits**	***p*-value**
	**n**	**%**	**n**	**%**			**n**	**%**	**n**	**%**			**OR (95% CI)**	
Assessment of alcohol consumption (via AUDIT-C)	46	7.2%	364	26.0%	5.39 (3.87; 7.50)	< 0.001	564	28.4%	1503	40.6%	2.63 (2.26; 3.05)	< 0.001	3.20 (2.38; 4.29)	< 0.001
Complete brief advice (safest not to consume and potential risks)	45	7.1%	263	18.8%	3.72 (2.66; 5.22)	< 0.001	371	18.7%	986	26.7%	2.07 (1.78; 2.41)	< 0.001	1.91 (1.43; 2.54)	< 0.001
Advice safest not to consume	87	13.7%	465	33.3%	3.88 (2.99; 5.03)	< 0.001	666	33.5%	1742	47.1%	2.62 (2.28; 3.01)	< 0.001	1.87 (1.43; 2.43)	< 0.001
Advice on potential risks	121	19.0%	393	28.1%	2.01 (1.59; 2.55)	< 0.001	539	27.1%	1249	33.8%	1.70 (1.49; 1.95)	< 0.001	1.32 (1.03; 1.70)	0.03
Complete care relative to level of alcohol risk (complete brief advice and referral)	43	6.8%	263	18.8%	3.92 (2.78; 5.53)	< 0.001	367	18.5%	984	26.6%	2.10 (1.80; 2.44)	< 0.001	1.92 (1.44; 2.56)	< 0.001
Assessment of alcohol consumption (via AUDIT-C) and complete care relative to level of alcohol risk	23	3.6%	175	12.5%	4.88 (3.10; 7.66)	< 0.001	251	12.6%	718	19.4%	2.32 (1.94; 2.76)	< 0.001	2.43 (1.70; 3.47)	< 0.001

*OR* Odds Ratio, *95% CI* 95% Confidence Interval; Intervention effects adjusted for sector, antenatal visit and time (month of antenatal visit); Missing 7 participants who did not provide all data for receipt of care measures

### Receipt of antenatal care addressing alcohol consumption during pregnancy by type of antenatal visit

As shown in Table 3, there were significant differences in intervention effectiveness between the initial antenatal visit and the 27–28 and 35–36 week gestation antenatal visits for all outcomes. The intervention effect for receipt of all guideline elements was greater for visits at 27–28 weeks gestation (OR: 3.43; 95% CI: 2.33–5.05) and 35–36 weeks gestation (OR: 4.88; 95% CI: 3.10–7.66) compared with the initial visit (OR: 1.64; 95% CI: 1.32–2.04). Despite the greater intervention effect, the proportion of women reporting receipt of all guideline elements relative to reported alcohol risk level at follow-up was lower for visits at 27–28 weeks gestation (13.3%) and 35–36 weeks gestation (12.5%) than at the initial visit (33.8%).

### Receipt of antenatal care addressing alcohol consumption during pregnancy by sector

As shown in Table 4, all outcomes were significant within each sector other than advice on potential risks in Sector Three. There were no significant differences in intervention effectiveness between the three sectors for any outcome.

**Table 4:** Receipt of antenatal care addressing alcohol consumption during pregnancy by sector

	**Sector 1 (urban)**						**Sector 2 (regional/rural)**					
**Model of care element**	**Baseline** ***N*** **= 1308**		**Follow-up** ***N*** **= 3175**		**OR** **(95% CI)**	***p*-value**	**Baseline** ***N*** **= 302**		**Follow-up** ***N*** **= 385**		**OR** **(95% CI)**	***p*-value**
	**n**	**%**	**n**	**%**			**n**	**%**	**n**	**%**		
Assessment of alcohol consumption (via AUDIT-C)	346	26.5%	1255	39.5%	2.55 (2.15; 3.03)	< 0.001	87	28.8%	182	47.3%	3.28 (2.27; 4.73)	< 0.001
Complete brief advice (safest not to consume and potential risks)	219	16.7%	791	24.9%	1.97 (1.65; 2.35)	< 0.001	65	21.5%	151	39.2%	2.78 (1.94; 3.97)	< 0.001
Advice safest not to consume	408	31.2%	1447	45.6%	2.55 (2.18; 2.99)	< 0.001	115	38.1%	219	56.9%	2.87 (2.04; 4.03)	< 0.001
Advice on potential risks	325	24.9%	1014	31.9%	1.62 (1.39; 1.89)	< 0.001	92	30.5%	182	47.3%	2.29 (1.65; 3.16)	< 0.001
Complete care relative to level of alcohol risk (complete brief advice and referral)	216	16.5%	789	24.9%	2.00 (1.67; 2.39)	< 0.001	65	21.5%	151	39.2%	2.78 (1.95; 3.98)	< 0.001
Assessment of alcohol consumption (via AUDIT-C) and complete care relative to level of alcohol risk	144	11.0%	562	17.7%	2.13 (1.73; 2.62)	< 0.001	46	15.2%	119	30.9%	3.13 (2.09; 4.67)	< 0.001
	**Sector 3 (regional/rural)**						**Between group differences**					
**Model of care element**	**Baseline** ***N*** **= 379**		**Follow-up** ***N*** **= 138**		**OR** **(95% CI)**	***p*-value**	**Sector 1 vs Sector 2**	**Sector 1 vs Sector 3**	***p*-value**			
	**n**	**%**	**n**	**%**			**OR (95% CI)**	**OR (95% CI)**				
Assessment of alcohol consumption (via AUDIT-C)	131	34.6%	66	47.8%	2.31 (1.46; 3.65)	< 0.001	1.28 (0.86; 1.91)	0.91 (0.56; 1.47)	0.40			
Complete brief advice (safest not to consume and potential risks)	87	23.0%	44	31.9%	1.79 (1.14; 2.82)	0.012	1.41 (0.95; 2.10)	0.91 (0.56; 1.48)	0.19			
Advice safest not to consume	143	37.7%	76	55.1%	2.73 (1.76; 4.22)	< 0.001	1.12 (0.78; 1.63)	1.07 (0.67; 1.70)	0.81			
Advice on potential risks	122	32.2%	53	38.4%	1.50 (0.99; 2.27)	0.059	1.41 (0.99; 2.01)	0.92 (0.59; 1.43)	0.14			
Complete care relative to level of alcohol risk (complete brief advice and referral)	86	22.7%	44	31.9%	1.82 (1.16; 2.87)	0.010	1.39 (0.94; 2.07)	0.91 (0.56; 1.48)	0.21			
Assessment of alcohol consumption (via AUDIT-C) and complete care relative to level of alcohol risk	61	16.1%	37	26.8%	2.33 (1.42; 3.83)	< 0.001	1.47 (0.94; 2.30)	1.10 (0.64; 1.87)	0.25			

*OR* Odds Ratio, *95% CI* 95% Confidence Interval; Intervention effects adjusted for sector, type of antenatal visit and time (month of antenatal visit); Missing 7 participants who did not provide all data for receipt of care measures

### Acceptability of the model of care addressing alcohol consumption during pregnancy

Of the 715 women who received at least one element of care in the follow-up period, 707 (98.8%) reported that the care received addressing alcohol consumption during pregnancy was acceptable. Ninety-nine percent of women who reported being asked about their alcohol consumption (586/589), being advised that it is safest not to consume alcohol during pregnancy (508/511) and being advised about the potential risks (376/378) reported that receipt of these individual care elements was acceptable. For Aboriginal women, reported acceptability was 95.5% (42/44) for the overall model of care, 100% (33/33) for being asked about their alcohol consumption, 96.9% (31/32) for being advised that it is safest not to consume alcohol during pregnancy and 100% (27/27) for being advised about the potential risks.

## Discussion

This is the first randomised controlled study internationally to estimate the effectiveness of a practice change intervention in improving the implementation of guideline recommended antenatal care addressing alcohol consumption during pregnancy. The intervention was effective in increasing the proportion of women who received an assessment of their alcohol consumption via a validated tool and care relevant to their alcohol risk level. Greater intervention effects were found for antenatal visits at 27–28 and 35–36 weeks gestation than at the initial antenatal visit for all primary outcomes. There were no differential intervention effects between the three sectors. Almost all women, including Aboriginal women, agreed that the model of care was acceptable.

The study findings support the limited evidence available regarding the effectiveness of implementation strategies in improving guideline recommended care addressing alcohol consumption during pregnancy. Like the Italian study [52], which based intervention on action research and training in Obstetrics and Gynaecology Units, we observed a positive effect of advice about consuming alcohol during pregnancy. The effect sizes of these two studies are not comparable as the Italian study reported receipt of ‘correct’ advice in a small sample of pregnant women who received information from their midwife, whereas our study reported on increases in receipt of advice in a large, random sample of women attending an antenatal visit. The effect sizes in our study are larger than the pooled effects of 32 studies included in a 2015 Cochrane review of tailored implementation interventions addressing determinants of health care practices in various clinical settings (OR: 1.56; 95% CI: 1.27–1.93) [52]. When comparing the effects of this intervention with those of the three individual studies in the review that explicitly reported use of an implementation framework or model [35–37], we found similar results. This suggests that the positive outcomes of the intervention may be attributable to the multi-strategy approach that was tailored to antenatal provider’s barriers and guided by the TDF. To understand the mechanisms by which implementation strategies affected study outcomes, process outcomes like antenatal providers’ exposure to, and perceived appropriateness of, the strategies need to be examined [33, 53].

Fewer than 20% of women at intervention follow-up received all elements of recommended assessment and care relevant to their alcohol risk level, which indicates that some elements may be harder for antenatal providers to implement into routine practice than others. The element of care least reported by pregnant women post-intervention was advice on the potential risks associated with alcohol consumption in pregnancy. Barriers such as a perception that women who have not disclosed alcohol consumption during pregnancy do not require an explanation of the risks [54], may persist for antenatal providers. Future research could assess the barriers specific to this care element to determine whether additional implementation strategies are required to support its provision. Additionally, an exploration of intervention effectiveness based on whether women reported consuming alcohol during pregnancy would further contextualise study outcomes and inform whether the tailoring of implementation strategies is required for clinicians seeing different groups of women [55]. The tailoring of strategies could potentially target the intervention to support the needs of different groups of pregnant women and facilitate efficiencies in providing alcohol assessment and care in time limited antenatal visits.

Greater intervention effects were found for outcomes at the 27–28 and 35–36 weeks gestation antenatal visits, which had low reported rates of care prior to the intervention. These outcomes demonstrate an important shift for maternity services because, although clinical guidelines recommend that alcohol consumption be addressed throughout the antenatal period, behavioural risk screening has previously been confined to the initial antenatal visit and not re-addressed unless a risk was identified [54, 56]. In the context of limited care at these later antenatal visits, the intervention supported practice change by providing a schedule for care and the supporting systems and resources. However, it also introduced a new task to these visits, which required time as well as new skills for some antenatal providers who may have not usually been the primary providers of this care. Further research that examines whether the practice change intervention was effective for all types of maternity services and antenatal providers at these visits is warranted to inform effective guideline implementation in public maternity services [55].

Despite the positive intervention effect, the proportion of women receiving guideline recommended care post-intervention remains less than optimal. The incremental cost effectiveness ratio of the practice change intervention has been estimated at $32,570 (95% CI: $32,566 - $36,340) per percent increase in women reporting receipt of the full guideline recommended model of care [57]. Often the results from implementation efficacy trials conducted in real-world settings are considered modest for the investment made [58]. It is increasingly recognised that ongoing, purposeful adaptations to implementation interventions may be required to maximise initial investments and optimise potential impacts [58]. Similar concepts are implicit in continuous quality improvement approaches often used in healthcare settings to enhance processes, safety and patient outcomes [59]. Such an approach could be applied with the public maternity services that participated in this trial to examine whether adapted strategies that are less comprehensive and less costly could further enhance the impacts of this trial.

It is also important to assess whether the organisational and system focussed strategies used in the intervention sustain improvements in care provision. In a 2015 systematic review of health professional’s adherence to clinical practice guidelines in medical care, only seven of 18 trials were found to have sustained practices one or more years after active implementation support ceased [60]. It is possible that common barriers to sustaining practice improvements in health service settings, including high staff turnover and workload pressures, may influence the ongoing provision of antenatal care addressing alcohol consumption in the participating maternity services [61]. If it is found that improvements have not been sustained, additional evidence-based sustainability strategies, such as continued training opportunities and systematic adaptations to the intervention to continually increase fit with service context, may be required to facilitate ongoing care provision [62].

The study findings should be interpreted in light of a number of strengths and limitations. First, the study design provided a number of pragmatic and scientific advantages, including receipt of the intervention by all maternity services and recruitment of like services that could act as their own control. The large sample size and length of data collection were additional strengths. Co-production by research team, maternity services and Aboriginal community was a strength as it engendered a novel intervention relevant to needs of the services and the women. A potential limitation of the study was that several outcome measures required women to recall specific information from the antenatal visit; however, we sought to minimise recall bias by conducting interviews/online questionnaires within four weeks of visits. A quantitative approach was used to assess women’s acceptability of the model of care, which may have limited women’s ability to fully express their views on the care that they received. Future research could seek to contextualise acceptability further by incorporating qualitative research approaches. The study was conducted within one local health district in Australia and thus, the extent to which the results can be generalised is unknown. However, as the model of care was based on evidence and clinical guidelines, the practice change intervention was developed to address barriers that are consistent with the literature, and study outcomes were not significantly different between urban and regional/rural sectors, there is potential that the intervention could be applied in other jurisdictions and achieve similar outcomes.

## Conclusion

The multi-strategy practice change intervention was effective in improving the implementation of guideline recommended care addressing alcohol consumption during pregnancy. Future research could explore the characteristics associated with improved care to inform whether further tailoring of the implementation strategies is required for different groups of pregnant women or maternity services. Additionally, an assessment of the study outcomes over time would determine whether care has been sustained and inform the need for additional sustainability strategies. Alcohol consumption in pregnancy is common and harmful and these results have important implications for public maternity services seeking to achieve positive outcomes for pregnant women and their babies.

## Data Availability

The datasets used and/or analysed during the current study are available from the corresponding author on reasonable request.

## Abbreviations

AHW: Aboriginal Health Worker
AMIHS: Aboriginal and Maternal Infant Health Services
AUDIT-C: Alcohol Use Disorders Identification Test – Consumption
CI: Confidence Interval
CME: Clinical Midwife Educator
HNELHD: Hunter New England Local Health District
NSW: New South Wales
OR: Odds Ratio
RR: Risk Ratio
TDF: Theoretical Domains Framework
